# Acupuncture for Visceral Pain: Neural Substrates and Potential Mechanisms

**DOI:** 10.1155/2014/609594

**Published:** 2014-12-29

**Authors:** Shuping Chen, Shubin Wang, Peijing Rong, Junying Wang, Lina Qiao, Xiumei Feng, Junling Liu, Jianliang Zhang

**Affiliations:** ^1^Department of Physiology, Institute of Acupuncture and Moxibustion, China Academy of Chinese Medical Sciences, Beijing 100700, China; ^2^Department of Acupuncture, China General Meitan Hospital, Beijing 100028, China

## Abstract

Visceral pain is the most common form of pain caused by varied diseases and a major reason for patients to seek medical consultation. Despite much advances, the pathophysiological mechanism is still poorly understood comparing with its somatic counterpart and, as a result, the therapeutic efficacy is usually unsatisfactory. Acupuncture has long been used for the management of numerous disorders in particular pain and visceral pain, characterized by the high therapeutic benefits and low adverse effects. Previous findings suggest that acupuncture depresses pain via activation of a number of neurotransmitters or modulators including opioid peptides, serotonin, norepinephrine, and adenosine centrally and peripherally. It endows us, by advancing the understanding of the role of ion channels and gut microbiota in pain process, with novel perspectives to probe the mechanisms underlying acupuncture analgesia. In this review, after describing the visceral innervation and the relevant afferent pathways, in particular the ion channels in visceral nociception, we propose three principal mechanisms responsible for acupuncture induced benefits on visceral pain. Finally, potential topics are highlighted regarding the future studies in this field.

## 1. Introduction

Pain is a major problem in clinic and a common cause to seek physician consultation. According to World Health Organization, over one-fifth of the world population has experienced some degree of chronic pain [1]. Visceral pain, as it literally means, is the pain originating from the internal (thoracic, pelvic, and abdominal) organs. Unlike somatic or neuropathic pain which is sharp and well-defined with clear demarcation, visceral pain has some unique characteristics such as being diffusely localized, frequently not being linked with viscera injury, usually being referred to other tissues and locations, and often being associated with marked negative affective reactions including pallor, profuse sweating, nausea, gastrointestinal disturbances, and cardiovascular changes [2]. Although some of visceral pain states are not lethal, they produce a considerable negative impact on daily lives, cause huge economic burden, and create tremendous pressure on the healthcare systems worldwide.

The pathophysiology of the visceral pain is extremely complex. Currently, analgesics (opiates, nonsteroidal anti-inflammatory drugs, and benzodiazepine), antispasmodics, antidepressants, and so forth are the most common medications for acute as well as chronic visceral pain conditions; yet they are not always optimal for the prominent adverse effect like addiction and constipation. Furthermore, the paradoxical development of analgesic tolerance, inadequate pain relief, and nociceptive sensitization with prolonged opioid use has also proved an unfortunate obstacle for their clinic applications [3–5].

Acupuncture has long been used to treat a variety of pathological disorders in China and the neighbor countries and comes to be recognized as a promising alternative therapy by the western medical community in recent years. Since 1950s, a large number of studies have been carried out in elucidating the mechanism behind acupuncture for varied types of pain including visceral pain, showing that acupuncture inhibits pain via some bioactive substances chiefly the opioids, which desensitize peripheral nociceptors and reduce the proinflammatory cytokines peripherally and depress the neuronal activities centrally, and serotonin and norepinephrine, which decrease the spinal N-methyl-D-aspartate receptor subunit GluN1 phosphorylation [6]. Additionally, given the fact that pain can be relieved by various modes of physical procedures such as sound, flicker, heat, vibration, and electricity, suggesting that one sensation can be suppressed by another, it is proposed that acupuncture analgesia is at least partly a result of signals' interaction and integration at different level of neuraxis, from dorsal root ganglion (DRG) to cortex, of afferents originated from acupoints and injured somatic and/or visceral sites [7, 8]. Further, the afferent inputs of somatic and visceral origins can, respectively, modify the activities of the converging neurons in the spinal cord or higher centers and ultimately modulate their information processing [9–11]. In this paper, we summarize the neural substrates of visceral nociception including the sensory innervation, the afferent pathways to the spinal cord and brain, and, in particular, the special channels in sensing different stimuli and mediating their cellular processes and thereby address the potential mechanisms behind acupuncture for the management of visceral pain. We hypothesize the following: (1) signals from the acupoints by acupuncture signals interact in DRG, spinal cord, and supraspinal structures of “pain matrix” with that from the injured internal organ(s) and thereby depress or even abolish the sensory (nociceptive) transmission and perception; (2) acupuncture can modulate the synthesis and secretion of endogenous components derived from enterocytes and more specialized cells like enteroendocrine and immune cells in intestinal epithelium via regulating the efferent activities of the spinal and supraspinal autonomous nerve centers and therefore is capable of pain modulation; (3) gastrointestinal (GI) microbiota act on the nociceptors via release of enteroendocrine or immune-derived mediators. The microbial products can probably excite the sensory endings (e.g. activation ions of TRPs) to directly evoke visceral pain. Acupuncture analgesia may be through regulation of GI motility and secretion which leads to reduction of pronociceptive substances by the GI microbiota. Considering the wide range of viscera from the higher esophagus, lung, heart, and GI tract to the lower pelvic organs, we hereon focus on the GI part for its general representativeness in visceral disorders.

## 2. Neural Substrates for Visceral Innervation

Visceral sensory neurons activate reflex pathways that control gut function and meanwhile give rise to important sensations such as fullness, nausea, and pain. As known, the internal organs are insensitive to cutting and burning but usually sensitive (perceived as nausea and painful) to ischemia, inflammation, and stretch or change of intense force by distension or contraction, and, in pathological conditions, less stimuli are usually required to induce the sensations [12, 13]. Different from the somatic sensory afferents, which arise only from the neurons located in DRG, the neurons innervating visceral structures from the esophagus to the transverse colon are distributed not only along DRGs of the respective segments, but also from the jugular and nodose ganglia [14, 15]. Notably, visceral structures distal to the transverse colon, particularly the distal colon, rectum, and bladder, are also innervated by two populations of afferents arising from two different levels of the spinal cord, that is, thoracolumbar and lumbosacral regions [16–18]. It should be mentioned that GI tract is regulated by both intrinsic and extrinsic components of the autonomic nervous system. Intrinsic innervation is controlled by the enteric nervous system (ENS), which consists of connecting nerve plexuses that extend between the muscular layers and the submucosa of the gut wall, and regulates GI motility, secretion, and absorption whereas it plays no major role in pain transmission. The extrinsic innervation of GI includes efferent parasympathetic as well as sympathetic pathways that are involved in the modulation of ENS activities [19, 20].

Visceral nociceptors, unlike their somatic counterparts, have no obvious anatomical specialization of the endings in the afferents that allow them to differentially respond to the various stimuli. For the case of GI tract, however, based on the distinct locations of the nerve endings in the gut wall, five types of sensory endings have been identified, that is, (1) the “intraganglionic laminar” endings in myenteric ganglia, (2) the “mucosal” endings located in the subepithelial layer, (3) the “muscular-mucosal” afferents with mechanosensitive endings adjacent to the muscularis mucosae, (4) the “intramuscular” endings within the smooth muscle layers, and (5) the “vascular” afferents whose sensitive endings are primarily on the blood vessels. Part of “silent” afferents might be the subset of inexcitable “vascular” afferents, which can be switched on by ischemia and inflammation induced mediators. Some of these receptors are part of a modality specific transduction pathway involved in sensory signalling from the gut lumen to vagal afferent endings in the mucosa. Others, which are activated by substances derived from multiple cellular sources during ischaemia, injury, and inflammation, act in a synergistic way to cause acute or chronic sensitisation of the afferent nerves to mechanical and chemical stimuli [21, 22].

### 2.1. Vagal Afferents

Vagal afferents have low threshold to mechanical stimulation and serve to predominantly convey the physiological range of information. Though they are able to directly encode not painful but non-painful sensations such as hunger, satiety, and nausea, they are however highly involved in the modulation of nociception in the spinal cord and the brain [23, 24]. Vagal afferents terminate and form elaborate structures within GI wall and are often activated by the physiological levels of distension and during peristalsis. Studies show that there are two types of vagal ending attributable to the mechanosensory function. One is the intramuscular array (IMA) located in both circular and longitudinal muscle layers where vagal afferents branch extensively to run parallel with the smooth muscle nerve bundles; the other is intraganglionic laminar endings (IGLE), which is basket-like and is distributed around myenteric ganglia. They form a transduction site for mechanosensitivity and are probably involved in emotional and behavioural aspects rather than pain cognition [21, 25, 26].

### 2.2. Spinal Afferents

Generally, nociceptive afferents innervate viscera via the splanchnic nerves, the paired nerves carrying fibers of the autonomic nervous system (visceral efferent fibers) as well as sensory fibers from the organs (visceral afferent fibers), all of which are attributed to be sympathetic except for the pelvic portion, which belongs to parasympathetic fibers. Structurally, the visceral sensory afferents are mostly made up of small, thinly myelinated (A*δ*) or unmyelinated (C) fibers with low mechanical thresholds, enabling them to code normal physiological and noxious stimuli as well [27–29]. It has been defined that the visceral nociceptors are these primary afferent fibers with high threshold to mechanical stimuli in the healthy internal organs that innervate blood vessels either within or outside the internal organs' wall [30, 31]; that is, they are actually a form of vascular endings, whereas low threshold afferents innervate the muscle layers of the gut wall or the villi of the mucosa [22]. Visceral pain results from the excitation of spinal visceral afferents rather than the vagal afferents with a few exceptions. One example is the afferents from esophagus which directly transmits nociceptive signals from the squamous epithelium in response to acid [32]. This means that, contrary to the vagal counterparts, spinal afferents are able to encode the supraphysiological levels of noxious events [33].

### 2.3. Ascending Pathways

It has been well documented that the peripheral and central pathways innervating viscera project to the central nervous system via autonomic sympathetic and parasympathetic nerves [12, 34, 35]. The spinal visceral afferent neurons are polymodal and their central endings project into laminae I, II (outer part IIo), and V of the mediolateral spinal dorsal horn for over several segments, where their activities are synaptically transmitted to viscerosomatic convergent neurons that receive additionally afferent synaptic inputs from the skin and the deep somatic tissues of the corresponding dermatomes [36]. These spinal second-order neurons include two types of neurons, that is, the interneurons and the projection neurons. The viscerosomatic projection neurons within laminae I and V constitute the major afferents from the spinal cord to the brain [37]. These are two classic ascending pathways, that is, spinothalamic and spinoreticulothalamic tracts, which carry pain information to the thalamic nuclei and brainstem reticular formations, respectively, and the former is implicated to the sensory-discriminative aspects of the pain experience, whereas the latter may be more relevant to poorly localized pains. In addition, the recently found spinoparabrachial tract attracted attention because the output of this region provides for a very rapid connection with the amygdala, a region generally considered to process information relevant to the aversive properties of the pain experience. Particularly, the dorsal column pathway was believed to play a crucial role in the transmission of visceral nociceptive information [38–40]. It should be mentioned that the transmission of signals from visceral afferents to the spinal second-order neurons is modulated by the endogenous descending systems from the brain stem, which are further under the cortical control [41]. It should be mentioned that there are two modes of firing of thalamocortical neurons in the thalamic relay nuclei, tonic and burst firing, which are believed to reflect the divergent states of sensory signal transmission from the thalamus to the cortex and have been shown to modulate visceral pain [42, 43].

As for the vagal and pelvic afferents, which innervate from the esophagus to the colon and rectum, respectively, they project centrally to the nucleus of the solitary tract (NTS) of the brainstem and the sacral spinal cord [44–47]. From NTS, the vagal afferents project densely to the parabrachial nucleus (PB) in the pons, the dorsal raphe, and numerous forebrain sites including infralimbic and olfactory cortices, amygdala, hypothalamus, hippocampus and the thalamus (via PB) [48–50].

## 3. Receptors and Mediators

Recent studies have identified some ion channels that may confer modality-specific sensitivity of these visceral afferents. These include purinergic channels including P2X and P2Y families [51–53], the family of acid sensitive ion (ASIC) channels [54–57], protease activated receptor 2 (PAR2) [58–60], and members of the transient receptor potential (TRP) family which have recently been implicated to play critical role in visceral pain. Here, we highlight the role of TRPs and meanwhile introduce other relevant neurotransmitters like opioid substances and serotonin (5-HT).

### 3.1. TRP Family

TRP channels are a group of ion channels located mostly on the plasma membrane of numerous human and animal cell types. These ion channels are relatively nonselectively permeable to cations including sodium, calcium, and magnesium and considered as molecular sensors to tissue damage and inflammation. The widespread expression of TRP channels in both neuronal and nonneuronal tissues suggests their critical roles in physiological as well as pathophysiological cellular processes including pain sensation and modulation [61–65].

#### 3.1.1. TRPV1

TRPV1 is extensively expressed in the gastrointestinal tract and serves as an important regulator of GI motility and visceral hypersensitivity. It conducts inward current in response to protons, noxious heat, and exogenous vanilloid compounds like capsaicin, lipids, and the second messenger signal molecule diacylglycerol DAG and is also regarded as the sensor responsive to high temperature (>43°C), low pH (pH < 5.9), and inflammatory origin of pain. Once activated, a sensation of burning pain is perceived, along with the release of substance P and CGRP, which trigger the neurogenic inflammation process [66].

Evidence shows that the majority of visceral afferents are peptidergic which express TRPV1 [67–69]. Malin et al. [70] have physiologically and neurochemically characterized the mechanosensitive colon afferents and found that up to 87% of high threshold afferents were TRPV1-positive, whereas 87% of low threshold were TRPV1-negative. Furthermore, TRPV1 and TRPA1 have a high degree of coexistence in the visceral afferents, which means the mechanosensitive colon neurons and the vast majority of TRPV1-responsive afferents can also be potentiated by the TRPA1 agonist mustard oil.

Clinical observations found that the patients with GI hypersensitivity due to inflammatory bowel diseases have more TRPV1-positive nerve fibers in muscle, mucosal, and submucosal layers than those of the controls [51, 71]. Animal study via immunohistochemical approach also revealed that the TRPV1 was present in the mucosa, submucosa, myenteric plexus, and circular and longitudinal muscle layers of the large intestine [72]. In TRPV1 knockout mice, the symptoms of acid-induced esophagitis were markedly relieved [73]. And siRNA-mediated knockdown of TRPV1 diminishes spontaneous visceral pain in mice [74], whereas upregulation of TRPV1 expression and function by administration of the nerve growth factor (NGF) has been shown to promote pain in chronic pancreatitis [75].

#### 3.1.2. TRPA1

TRPA1 is a promiscuous chemical nocisensor that is also involved in noxious cold and mechanical sensation. It is present in a subpopulation of A*δ*- and C-fiber nociceptive sensory neurons as well as in other sensory cells including epithelial cells [76], responding to noxious cold, to exogenous compounds including mustard oil and cinnamaldehyde, to menthol and cannabinoids, and to endogenous products derived from plasma membrane including 4-hydroxynonenal and to inflammatory mediators such as H_2_O_2_ and 15-deoxy-delta(12,14)-prostaglandin J(2) [77–79]. In primary sensory neurons, ions like calcium and sodium flow through TRPA1 into the cell to induce a sequence of reactions including the membrane depolarization, action potential discharge, and neurotransmitter release both at peripheral and central neural projections. In addition to being activated by cysteine and lysine reactive electrophiles and oxidants, TRPA1 is indirectly activated by proinflammatory agents via the phospholipase C signaling pathway, in which cytosolic calcium is a crucial regulator of channel gating.

Just like TRPV1, TRPA1 is also expressed in visceral afferent neurons and plays an important role in visceral sensory transduction, particularly in the context of visceral inflammation and pain in both gastrointestinal and urinary tracts [80–82]. In rodent models of colitis generated by intracolonic infusion of 2, 4, 6-trinitrobenzene-sulfonic-acid (TNBS) and drinking dextran-sulfate-sodium-salt- (DSS-) containing water, the presence of intestinal inflammation mediated by TVPA1 via the substance P release has been demonstrated [83–86]. Furthermore, endogenously secreted inflammatory mediators, for instance, 4-hydroxynonenal (4-HNE), can activate TRPA1 to initiate a vicious positive feedback cycle [85, 87]. It is interesting to mention that, however, one most recent study showed that selectively activating TRPA1 leads to constipation and abdominal pain relief, which are assumed to be arising from activation of vagus nerves from the mucosal side of the gut wall and alteration of the systemic blood flow, respectively [88].

#### 3.1.3. TRPV4, TRPM8, and TRPC4

It has been reported that the mechanosensitive TRPV4 is also expressed in visceral sensory DRG neurons, and its agonists evoke visceral hypersensitivity, which is attenuated by TRPV4-targeted gene knockdown or knockout [89, 90]. TRPV4-mediated visceral hypersensitivity is enhanced by histamine, 5-HT, and activation of PAR2 [91, 92]. In addition to the visceral sensory DRG neurons, TRPV4 is also found in the urothelial cells and inhibition of TRPV4 by pharmacological or genetic ablation improves the bladder overactivity of mice [93, 94].

The presence of cooling sensing TRPM8 in colonic DRG neurons has been confirmed. TRPM8 expressed in high threshold sensory neurons may couple to TRPV1 as well as TRPA1 and thereby inhibits their downstream chemosensory and mechanosensory function as TRPM8 activation blocks TRPV1-mediated CGRP release and attenuates inflammatory response [95, 96].

TRPC4 has been implicated in the tissue-specific and stimulus-dependent regulation of intracellular calcium signaling. Study shows that rats with a transposon-mediated TRPC4-knockout mutation displayed tolerance to visceral pain induced by colonic mustard oil exposure, rather than the somatic or neuropathic pain stimuli. Moreover, wild-type rats treated with a selective TRPC4 antagonist (ML-204) prior to mustard oil exposure mimicked the behavioral responses of the TRPC4-knockout rats in a dose-dependent manner, suggesting that TRPC4 is crucial for detection and/or transmission of inflammatory colonic visceral pain sensation [97].

### 3.2. Miscellaneous

#### 3.2.1. Opioid

Opiates are powerful drugs to treat severe pain via acting three opioid receptors, that is, *μ*, *δ*, and *κ*, which are distributed at the central and peripheral sites within the pain control circuits of the nervous system. For GI tract, studies show that, in the injured area (e.g., inflamed), there are T (e.g., T-helper 1 and Th17) cells accumulated with high level of opioid substances leading to a significant reduction of the visceral pain and hypersensitivity, whereas, in the control mice, the macrophages and epithelial cells did not secret opioids [98, 99]. It has been shown that only *κ*, rather than *μ* or *δ* opioid receptor agonists, effectively decreases the activation of colorectal afferents by mechanical distension [100]. Recent data indicate that visceral nociceptors increase their expression of *κ* receptors in a model of chronic visceral hypersensitivity and, accordingly, their inhibition by *κ* antagonists probably underlies the clinical efficacy of pain relief in IBS victims [101, 102].

#### 3.2.2. Serotonin

Serotonin (5-HT) is one of the most abundant molecules in the GI tract, playing a crucial role in physiological (e.g., motility, secretion, and visceral sensitivity) as well as pathological functions like immune and inflammatory responses. It is estimated that approximately 95% of the human body's 5-HT is produced and stored in enterochromaffin (EC) cells in the intestinal epithelium [103, 104]. The wide range of pathophysiological actions exerted by 5-HT is mediated by several different serotonergic receptor types and subtypes such as 5-HT1, 5-HT2, 5-HT3, 5-HT4, and 5-HT7 that are expressed in the intestine. It has been demonstrated that administration of 5-HT3 receptor antagonist brings about therapeutic benefits in alleviating pain and other symptoms of IBS patients [105, 106]. In addition, activation of 5-HT4 receptor has led to promising results with symptom relief of constipation IBS victims [107].

#### 3.2.3. Adenosine Triphosphate- (ATP-) Gated Ion Channels

ATP-gated ion channels have been identified in afferent nerve endings of the large intestine where they respond to the nociception due to other pathological conditions (e.g., inflammation, infection, and cell lesion) of the digestive tract [108]. There are two types of receptors: one is ATP-gated P2X (also known as purinergic) and the other is G-protein coupled P2Y. Evidence indicates that, in chronic visceral hypersensitivity, there is upregulation of P2X3 receptor expression, the subgroup of P2X receptor relevant to this pathological process in rat colons [53]. Further study by intracolonic administration of zymosan which causes hypersensitivity in the absence of inflammation verified the crucial role of peripheral and central P2X3 receptors in the mediation of the colonic hypersensitivity in rats [109]. In addition, study revealed that P2X7 receptor, another subgroup of P2X receptors which is located in the macrophages, is one of the potential mediators for pain and inflammation via the interleukin- (IL-) 1b [110].

#### 3.2.4. Somatostatin

Somatostatin (SST) is a peptide hormone that regulates the endocrine system and affects neurotransmission and cell proliferation via interaction with G protein-coupled SST receptors and inhibition of the release of numerous secondary hormones. In addition to the hypothalamus of the brain, SST is mainly secreted in the GI system including stomach, intestine, and *δ* cells of the pancreas. Generally, SST exerts inhibitory effects on secretion and motility of GI tract [111]. However, there is evidence showing its inhibition on the visceral perception as activation of SST receptor leads to reduction of the afferent firing in the “wide-dynamic range” fibers, which is involved in noxious information transmission [112, 113]. Furthermore, mice deficiency of SST has been demonstrated to have elevated responses to both low and high threshold distension as well as chemical stimulation, suggesting a tonic inhibition by SST on the visceral sensitivity [114].

## 4. Management of Pain and Visceral Pain by Acupuncture

From the point of traditional Chinese medicine (TCM) view, many diseases including pain and visceral pain develop due to the stagnation of* qi* and* blood* along the meridians throughout the body. As stated in* The Yellow Emperor's Inner Canon* (*Huang Di Nei Jing*), an ancient medical book compiled around 770–221 B.C., “pain comes from meridian stasis, and goes away in smooth circulation” [115]. Accordingly, it is believed in TCM that acupuncture achieves its efficacy like pain relief via regulation of* qi* and* blood* and promotion of their circulation along the meridians [116].

### 4.1. Clinical Observations

In the past decades, a large number of studies have proved that acupuncture induces analgesic effects, known as acupuncture analgesia in both humans and animals. Clinic observations have shown that acupuncture is quite effective in relieving chronic pain which can benefit 50–85% of patients [117, 118]. An original test on healthy participants by Chinese investigators demonstrated that acupuncture manipulation of “Hegu” (LI4) acupoint (the 4th point along large intestine meridian with anatomical location on the dorsum of the hand between the 1st and 2nd metacarpal bones and innervated by the superficial ramus of the radial nerve, the most often used acupoint for head and facial diseases) can gradually elevate the pain threshold and attain to the climax usually after 20–40 min of needling which persists for over 30 min after terminating the manipulation. Administration of 2% procaine into the acupoint prior to acupuncture intervention abolished this effect, suggesting that the adjacent nerves and/or nerves' endings take crucial role in this process [119, 120]. To examine the role of neurotransmitters in this inhibitory effect, the cerebrospinal fluid from acupuncture-treated rabbits was infused into recipient rabbits and the analgesic effect reproduced in the latter as expected, indicating that acupuncture-induced analgesia might be mediated by some bioactive substances released in the cerebrospinal fluid [121]. Since then, acupuncture analgesia was repeatedly verified by investigators from different labs [122–125].

In a pilot study, Xing et al. [126] demonstrated that transcutaneous electrical acustimulation (TEAS), a procedure similar to acupuncture stimulation, at “Zusanli” (ST36) acupoint and “Neiguan” (P6) acupoint, notably increased the threshold of rectal sensation of defecation and pain of patients with irritable bowel syndrome (IBS). Further, in a preliminary, randomized, well-controlled trial, twenty-nine IBS subjects were randomized to either treatment group or control group. The Clinical Global Impression Scale was administered before intervention to establish baseline severity and on completion of the 4-week, eight-session treatment intervention. After 4 weeks (twice per week) of treatment, the average daily abdominal pain/discomfort improved significantly whereas the control group showed minimal reduction. In addition, the intestinal gas, bloating, and stool consistency composite score exhibited a similar pattern of improvement [127]. For postoperative pain due to visceral surgery, acupuncture can effectively reduce the pain, lower analgesic consumption, and improve other symptoms like nausea and vomiting [128]. Interestingly, a study conducted in healthy subjects showed that acupuncture transcutaneous electrical nerve stimulation, a noninvasive modality of procedure, could enhance the perception (distention, defecation, discomfort, and pain) threshold in comparison with the placebo controls. More participants in acu-TENS group tolerated the painful colorectal distension stimulus (>40 mmHg) than placebo-TENS group. Concomitantly, the plasma *β*-endorphin level of subjects in acu-TENS group was significantly elevated comparing with that of the placebo-TENS group [129, 130].

In this connection, it is interesting to mention that by measuring the pressure-pain threshold, we found that in patients with gastric ulcer or gastritis, there are some tender points distributing around the abdomen and the back, overlapping the acupoints such as “Burong” (ST19), “Liangmen” (ST21), “Weishu” (BL21), and “Pishu” (BL20), which means that the acupoints might be sensitized in pathological states [131]. This coincides with the earlier finding that there is high degree of correspondence in response properties and spatial locations between acupoints and tender points to visceral pain [132]. Also intriguingly, spinal cord stimulation (SCS), a therapy which uses device to generate electrical pulses to the spinal cord, produces remarkable effects in control of refractory pain. One recent study by Baranidharan et al. [133] showed that ventral spinal column stimulation led to significant reduction of the visual analog pain scores and the opioid consumption and improvement of quality of life in patients with visceral neuropathic pain. We assume that, in terms of impact on the spinal neurons and tracts, the “endogenous” impulses evoked by acupuncture or electroacupuncture (EA) stimulation are equivalent to those of SCS, which is in fact an “exogenous” pattern resulting from the electric current.

### 4.2. Animal Studies

#### 4.2.1. Electrophysiological Mechanism

Apart from the clinic studies, numerous laboratory experiments have been carried out to evaluate the acupuncture benefits for visceral pain in animals and further explore the underlying mechanisms [125, 134, 135]. Early investigation by Guoxi [136] showed that electric stimulation of ST36 acupoint or the somatic nerve could inhibit the visceral origin nociceptive neuronal activities of the posterior portion of thalamus in cat. Work performed in our laboratory revealed the effects on nociception by acupuncture. With the help of the extracellular recording technique, we analyzed the neuronal activities in nucleus gracilis in the low medulla of anesthetized rats. It was showed that all forty-three neurons responsive to colorectal distension (CRD) had excitatory responses to tactile stimuli of their receptive fields (RF). Interestingly, their tactile responses were predominantly (31/43 units) enhanced by preceding CRD, and, conversely, the neuronal responses to CRD were reduced in 22/43 units when preceded by tactile stimulation [137]. Subsequently, we assessed the neurons of ventroposterior lateral thalamus, the higher center for sensory information processing in the brain, to examine the somatovisceral integration and the acupuncture effects. The results demonstrated that, among numerous neurons responding to tactile stimulation, seventy-two units were found responsive not only to innocuous stimulation on skin RF (60 activated, 12 inhibited) but also to noxious CRD. Electrical stimulation (2 Hz of frequency, 1 mA of intensity) of the neuronal somatic receptive field center reduced the subsequent neuronal responses to CRD in 40 neurons tested. Further, high frequency stimulation (100 Hz) produced stronger inhibition than low frequency (2 Hz) stimulation at RF. Our data suggest that somatovisceral interactions and integrations take place at multiple levels in the dorsal column-medial lemniscus system [138]. Rong et al. [139] reported that EA remarkably inhibits CRD induced discharges of lumbar spinal dorsal horn neurons and, notably, this effect was abolished by blockade of the central descending pathway with ice between the cervical and thoracic portion. In the latest study, Liu et al. [140] showed that EA stimulation inhibited the excitatory neurons in nucleus tractus solitarius (NTS) by CRD in rats, indicating its involvement in the mediation of acupuncture analgesia on visceral pain. Altogether, these data suggest that acupuncture depresses nociceptive ascending visceral signals in varied structures of different neuraxis.

#### 4.2.2. Opioid Mechanism

Central and peripheral chemical components have been proved to take predominant role in acupuncture induced analgesia. Among numerous neurotransmitters (modulators) and biological substances, opioid peptides are prominent for their powerful analgesic action and the finding of the endogenous opioids system imposed a significant impetus to the delineation of the work mechanism behind acupuncture [124, 141]. It was reported that the stress-induced visceral hypersensitivity in rats could be alleviated by EA. And this effect was blocked by pretreatment with naloxone or was completely reversed by administration of m-naloxone, a peripherally restricted opioid antagonist. Study via patch clamp showed that EA treatment normalized the enhanced excitability of colon DRG neurons and, further, in vitro application of [D-Ala(2), N-MePhe(4), Gly(5)-Ol] enkephalin (DAMGO) suppressed the enhanced excitability of colon neurons from rats with chronic visceral hypersensitivity (CVH) [135, 142]. These findings suggest that EA induced analgesia might be largely mediated by endogenous opioid pathway.

#### 4.2.3. 5-HT Mechanism

Other substances like 5-HT are also reported to participate in acupuncture analgesia [8, 143]. 5-HT hyperactivity has been reported to be associated with visceral hypersensitivity in patients with IBS, a role that was further supported by the effectiveness of 5-HT3 receptor antagonist for the treatment [144, 145]. Wu et al. [146] evaluated the effect of EA in treating visceral hyperalgesia of rats due to neonatal maternal separation stress. They found that EA significantly enhanced the pain threshold and reduced the visceromotor response compared to those in sham acupuncture group. Additionally, EA significantly suppressed Fos expression in dorsal raphe nuclei of brainstem, superficial dorsal horn of spinal cord, and colonic epithelium but suppressed 5-HT expression only in brainstem and spinal cord, indicating that EA attenuates visceral hyperalgesia through downregulation of central serotonergic activities in the brain-gut axis. Other studies revealed that EA at auricular points could increase, concurrently with the decrease of vasomotor responses, the mRNA expression of the 5-HT1a receptor in both the colon (peripheral) and raphe nuclei (central) in CRD induced visceral pain rats. By means of immunohistochemistry and spectrophotofluorometer detection, it has been demonstrated that EA could significantly increase the 5-HT(4a) and serotonin transporter expression whereas it could decrease 5-HT concentration in the colon tissue of CVH rats [147, 148]. Liu et al. [149] assayed the colon tissues of CVH rats via enzyme-linked immunosorbent assay (ELISA) and showed that EA reduced the 5-HT and increased the 5-HT4R concentration but had no effect on the 5-HT3R concentration. The evidence implicates the role of serotonergic component in acupuncture effects for visceral pain.

#### 4.2.4. Others

Our own data showed that acupuncture alleviates neuropathic hypersensitivity partially via the inhibition of COX-2 expression in the spinal cord [150]. Tian et al. [134] reported that EA reduced the increased expression of phosphorylated NMDA receptor subunit 1 (pNR1) in the lumbar spinal cord of CVH rats, implying the involvement of spinal NMDA receptors phosphorylation in this process. It is worth mentioning that the thriving molecular biotechnologies have facilitated the elaboration of acupuncture mechanism in transcriptional and posttranscriptional level. Gene expression is a subtle indicator of interaction between genome and stimulation and its profiling of particular regions is used to decipher the molecular changes in special function and behavior in response to environmental changes [151]. By means of gene microarray analysis, it has been revealed that sixty-eight genes were differentially expressed more than 2-fold in the spinal nerves of neuropathic model rat in comparison to the normal and restored to the normal expression level after the EA treatment. These genes are involved in a number of biological processes, including the signal transduction, gene expression, and nociceptive pathways [152]. For hypothalamus, the transcriptional profile was that, during acupuncture analgesia, sixty-three and three genes were up- and downregulated, respectively. Half of the differentially expressed genes were classified to be involved in ion transport, sensory perception, synaptogenesis and synaptic transmission, signal transduction, inflammatory response, and so on. For dorsal horn of spinal cord, the expression of the neurotransmitter system related genes was upregulated significantly in the higher responders rats to acupuncture while the proinflammation cytokines related genes in nonresponder rats were upregulated more significantly than that in higher responders rats after 2 Hz and 100 Hz EA stimulation, especially in the case of 2 Hz stimulation [153]. It is supposed that these differentially expressed genes might be the new targets for nociceptive study and potential pharmaceutical interventions [154]. Interestingly, in addition to genes, acupuncture could alter the cellular and enzymatic activities of diffuse tissues. In healthy rats, for instance, EA stimulation of “Zusanli” acupoint (ST36) increased natural killer cell activity in the spleen by approximately 44% and the activity of superoxide dismutase in the hypothalamus, liver, and red blood cells. These findings indicate that acupuncture brings about efficacy via various ways including cellular activity, genes network, and metabolic enzymes in the body [155].

## 5. Conclusion and Future Considerations

Acupuncture achieves analgesic effects on visceral pain via multiple modes (signal interaction and integration at neurons, ion channels, activation of central descending inhibition pathways, gene regulation, etc.) and recruitments of a variety of biochemicals (opioids, 5-HT, NMDA, etc.). For future study, it is interesting to probe the effects of acupuncture on GI microbiota as the latest evidence indicated that bacteria directly activate nociceptors and thereby trigger pain and visceral pain without mobilizing other mediators (B cells, T cells, etc.) in inflammatory mice [156]. We also suggest the future animal investigations be performed in accordance with the “theory of correlation between somatic acupoints and corresponding internal organs” in traditional Chinese medicine. Moreover, it is important to optimize the visceral pain types with acupoints prescription and stimulation parameters for better therapeutic benefits.
